# Durability of HIV viral suppression in South Africa: a national cohort study

**DOI:** 10.1016/S2352-3018(26)00079-2

**Published:** 2026-08

**Authors:** Evelyn Lauren, Jacob Bor, Trevor Graham Bell, Dickman Gareta, Khumbo Shumba, Mazvita Muchengeti, William MacLeod, Matthew P Fox, Wendy Stevens, Tshepo Molapo, Musa Manganye, Dorina Onoya, Koleka Mlisana

**Affiliations:** Health Economics and Epidemiology Research Office, Department of Internal Medicine, School of Clinical Medicine, Faculty of Health Sciences, University of the Witwatersrand, Johannesburg, South Africa (E Lauren BS, J Bor SD, K Shumba MSc, W MacLeod SD, M P Fox PhD, D Onoya PhD); Department of Biostatistics, Boston University School of Public Health, Boston, MA, USA (E Lauren); Department of Global Health, Boston University School of Public Health, Boston, MA, USA (J Bor, W Macleod, M P Fox); Department of Epidemiology, Boston University School of Public Health, Boston, MA, USA (J Bor, M P Fox); National Institute of Communicable Diseases, National Health Laboratory Service, Johannesburg, South Africa (T G Bell PhD, M Muchengeti PhD); Africa Health Research Institute, Somkhele, South Africa (D Gareta BS, J Bor); Institute of Social and Preventive Medicine, University of Bern, Bern, Switzerland (D Gareta); Graduate School for Health Sciences, University of Bern, Bern, Switzerland (D Gareta); National Health Laboratory Service, Johannesburg, South Africa (M Muchengeti, W Stevens MMed, K Mlisana PhD); Diagnostic Innovations Hub, University of Witwatersrand, Johannesburg, South Africa (W Stevens); HIV/AIDS and STIs Cluster, National Department of Health, Pretoria, South Africa (T Molapo PhD, M Manganye DrPH); School of Laboratory Medicine and Medical Sciences, University of KwaZulu Natal, Durban, South Africa (K Mlisana)

## Abstract

**Background:**

People with an HIV viral load less than 200 copies per mL cannot transmit HIV sexually, but viral rebound can lead to transmission risk. We aimed to quantify 12-month durability of viral suppression in South Africa’s national HIV programme.

**Methods:**

We conducted a retrospective observational cohort study to assess the 12-month durability of viral suppression in South Africa’s National Health Laboratory Service National HIV Cohort, a probabilistically linked, de-identified database including all viral loads in the public sector HIV programme. Individuals aged 15–59 years were followed up for 18 months from their first viral load less than 200 copies per mL occurring between July 1, 2021, and June 30, 2022 (baseline). Guidelines specified annual monitoring if viral load was less than 200 copies per mL. The first outcome of our study was viral monitoring at 12 months, defined as any viral load test 6–18 months after baseline. The second outcome of our study was 12-month viral load, categorised according to WHO transmission risk thresholds: less than 200 copies per mL (zero risk), 200–999 copies per mL (almost zero risk), and 1000 copies per mL or greater. We fit modified Poisson regression models to assess predictors of these outcomes.

**Findings:**

Our study included 3 584 203 individuals, reflecting all people living with HIV aged 15–59 years with an HIV viral load less than 200 copies per mL in the national HIV programme between July 1, 2021, and June 30, 2022. 2 588 294 (72·2%) individuals were female and 995 909 (27·8%) individuals were male. Of the 3 584 203 individuals with a baseline viral load less than 200 copies per mL, 2 858 992 (79·8%) were virally monitored at 12 months. Of those, 2 605 311 (91·1%) had a 12-month viral load less than 200 copies per mL, and 2755122 (96·4%) had a 12-month viral load less than 1000 copies per mL. Individuals with a previous history of viral suppression had the highest rates of monitoring (1 998 198 [87·2%] of 2 291 511 individuals) and continued suppression at less than 1000 copies per mL (1 950 857 [97·6%] of 1 998 198 individuals). Those least likely to remain virally suppressed included young men, young women, and people with previous elevated viral loads. Results were robust to linkage errors and different outcome definitions.

**Interpretation:**

People with viral load less than 200 copies per mL who remained on antiretroviral therapy still had zero or almost zero transmission risk 12 months later. People living with HIV with viral load less than 200 copies per mL can rely on Undetectable=Untransmittable as a prevention strategy, so long as they remain on antiretroviral treatment.

**Funding:**

US National Institutes of Health.

## Introduction

People living with HIV who take antiretroviral therapy (ART) and maintain a viral load less than 200 copies per mL cannot transmit HIV sexually.^1^ This conclusion, popularised by the slogan Undetectable=Untransmittable (U=U), is supported by evidence from several large cohort studies^2–5^ and has been endorsed by the US Centers for Disease Control and Prevention and WHO. A review concluded that risk of HIV transmission is zero at viral load less than 200 copies per mL and negligible or almost zero at viral loads of 200–999 copies per mL.^6,7^

The efficacy of U=U hinges on the extent to which individuals on ART can maintain viral suppression. Some clinicians and policy makers have expressed concern about viral rebound and fear that U=U might offer false assurance,^8^ particularly in settings where viral loads are routinely monitored only every 12 months. These concerns have delayed dissemination of U=U as a prevention tool, particularly in sub-Saharan Africa where most people living with HIV reside.^9–11^ Ironically, failure to provide information on the prevention benefits of ART could undermine motivations for long-term adherence, leading to greater transmission and hindering HIV control at the population level.^12^

Evidence is scarce on the 12-month durability of viral suppression among people on ART in sub-Saharan Africa (appendix pp 4–8).^13–18^ Clinical cohort studies have reported widely ranging rates of 12-month viral rebound, from 1·4%^14^ to 20·2%,^16^ leaving clinicians with substantial uncertainty as to the risk their patients face. Half of these studies were from the 2000s, when drug side-effects hampered adherence. All studies had relatively small sample sizes, were undertaken in specific clinical settings, and did not reflect the full diversity of experiences among people living with HIV. Furthermore, each study used a different definition of viral rebound, with none aligned to WHO’s HIV transmission risk categories.^6^

To inform dissemination of U=U in sub-Saharan Africa, we assessed 12-month durability of viral suppression relative to WHO transmission risk thresholds. We leveraged data from South Africa’s National Health Laboratory Service (NHLS) National HIV Cohort, including all viral loads from the country’s public sector HIV programme.

## Methods

### Study design and participants

South Africa has the world’s largest number of people living with HIV and the largest HIV treatment programme. HIV treatment is provided for free in the public sector, which provides 91% of ART nationally.^19^ Women comprise 69% of individuals on ART. Mass provision of HIV treatment has raised life expectancy by over a decade in South Africa.^20^ However, one in three people living with HIV remain virally unsuppressed, leading to about 170 000 new infections annually.^21^ HIV care follows national guidelines for diagnosis, ART initiation, choice of regimen, and laboratory monitoring to assess treatment efficacy. South Africa’s 2019 ART guidelines replaced efavirenz with dolutegravir in first-line therapy,^22^ and 69% of individuals initiated dolutegravir between 2019 and 2022.^23^

The NHLS conducts all viral load testing for South Africa’s public sector HIV treatment programme and archives all test results in a centralised database. In previous work, we created a unique person identifier via probabilistic record linkage, enabling analysis of the NHLS database as a National HIV Cohort.^24^ In validation, the linkage algorithm showed high accuracy, with a 1% overmatching rate and a 6% undermatching rate.^25^ The NHLS National HIV Cohort, including data from Jan 1, 2004, to Dec 31, 2023, allows assessment of individual-level HIV care trajectories in the public sector, regardless of transfer between facilities.^26^

We conducted a retrospective observational cohort study using de-identified data from the NHLS National HIV Cohort. The dataset included: anonymised person identifier, sex, age, viral load test date, viral load test result, type of health facility that ordered the test, and province of the health facility. The cohort was assembled from routinely collected NHLS laboratory data and comprised all individuals undergoing laboratory testing as part of routine clinical HIV care. Approval for the study, including a waiver of consent, was obtained from University of Witwatersrand Human Research Ethics Committee (M200447), Boston University Medical Campus Institutional Review Board (H-31968), and NHLS Academic Affairs and Research Management System Review Committee (PR2010539). Representatives of the NHLS and National Department of Health—both public institutions—were involved in the design and interpretation of the study; there was no direct patient involvement.

### Study population and data collection

We defined the study population as all people living with HIV aged 15–59 years who had a viral load of less than 200 copies per mL between July 1, 2021, and June 30, 2022 (study entry period), in South Africa’s public sector HIV programme. No sampling was conducted, as this was a complete population study. Individuals aged 60 years and older were excluded to minimise the risk of death during follow-up. We additionally excluded individuals who were younger than 15 years and individuals with missing or unknown sex, age, or facility at baseline. All viral load measurements from public sector clinics and hospitals were included; individuals with viral load measurements at other facility types (eg, prisons or military bases) were excluded from the analysis. Individuals were followed up for 18 months, beginning on the date of their first viral load less than 200 copies per mL in the study entry period. We refer to this date as the study baseline. We obtained viral load histories for all individuals dating back at least 5 years. Sex (male or female) was recorded by clinicians on the patient’s blood sample for the baseline viral load. Information on race or ethnicity was not routinely captured and was not included in the analysis.

### Outcomes

The first outcome of our study was viral monitoring at 12 months. South African national guidelines recommended annual viral load monitoring for individuals established on ART and virally suppressed.^22^ The first viral load test was performed 6 months after ART initiation; if an individual was virally suppressed, they would have another viral load test at 12 months, followed by annual viral load monitoring for those who remained suppressed. The presence of previous viral load tests served as a proxy for duration on ART. Guidelines specified more frequent viral load monitoring for individuals with viral load higher than 200 copies per mL; however, these individuals were excluded from our sample. We defined monitored at 12 months as having any viral load test within the window 6–18 months after baseline. If a person had multiple viral load measurements within this window, the earliest measurement was chosen for analysis (in sensitivity analysis, we expanded the range to 4–18 months after baseline). Per guidelines, we assumed that all individuals with a 12-month viral load test were accessing HIV care and receiving ART, although some of these individuals might have had treatment interruptions between baseline and their 12-month viral load test. Individuals without a 12-month viral load test might have dropped out of care, died, transferred to the private sector, migrated out of South Africa, or be receiving care but not virally monitored.

The second outcome of our study was durability of viral suppression. Individuals with a 12-month viral load test were classified into three WHO-defined HIV transmission risk categories: viral load less than 200 copies per mL (zero risk), viral load of 200–999 copies per mL (almost zero risk), and viral load of 1000 copies per mL or greater (elevated risk).^7^ We defined viral rebound as an indicator for 12-month viral load of at least 200 copies per mL after a baseline viral load less than 200 copies per mL. We defined durability of viral suppression as whether a person with viral load less than 200 copies per mL had a viral load less than 200 copies per mL or less than 1000 copies per mL at 12 months.

We analysed these outcomes alongside clinical and demographic factors. We categorised individuals based on their viral load history—ie, viral loads occurring before their baseline viral load less than 200 copies per mL. Individuals whose baseline viral load was their first ever viral load test were classified as first viral load and suppressed. These individuals likely reached viral suppression within 6 months of ART initiation. Individuals who had previous viral load tests before baseline and whose most recent viral load was less than 200 copies per mL were classified as previous viral load and suppressed. These individuals were further stratified based on the number of years (1, 2, 3, 4, and ≥5) they were continually suppressed. Finally, individuals whose last previous viral load before baseline was 200–999 copies per mL or 1000 copies per mL or greater were classified as previous viral load and unsuppressed. We stratified analyses by age (15–24 years, 25–34 years, 35–44 years, and 45–59 years), sex (female *vs* male), baseline facility type (clinic *vs* hospital), urbanicity (urban, peri-urban, or rural), and by South Africa’s nine provinces.

### Statistical analysis

We calculated the percentage of individuals monitored at 12 months and the percentages of monitored individuals who had 12-month viral loads in different categories: less than 200 copies per mL, 200–999 copies per mL, and 1000 copies per mL or greater. We estimated these quantities for the full study population and stratified by individuals’ viral load history, age, sex, urbanicity, and province. We suppressed 95% CIs because there was no sampling variability in this complete population analysis and the population size is so large that confidence intervals do not provide meaningful additional information beyond the point estimates.

To assess predictors of 12-month viral monitoring and viral rebound among monitored individuals, we fit modified Poisson regression models^27^ with indicators for age, sex, facility type, viral load history, and province as predictors. Modified Poisson regression with heteroske-dasticity-robust SEs provides valid inference for relative risks, even where the equal variance assumption is violated. For each outcome, we estimated crude risk ratios (RRs) with asymptotic 95% CIs and adjusted RRs (aRRs) in models adjusting for all other predictors.

Finally, to assess changes in the durability of viral suppression over time, we replicated our analysis using data from the NHLS National HIV Cohort from 2015 to 2018, before the roll-out of dolutegravir and the COVID-19 pandemic.

We conducted several sensitivity analyses. We excluded individuals whose baseline viral load was tested in a hospital to minimise the share of individuals tested for reasons other than routine monitoring (most HIV care is provided in primary health clinics^28^). We expanded the definition of monitored at 12 months to include any viral load test within the window 4–18 months after baseline, capturing individuals with a viral load earlier than scheduled. We restricted the population to individuals with a baseline viral load less than 50 copies per mL, the more conservative threshold used in South African guidelines to define an undetectable viral load. Finally, we restricted the data to individuals in the NHLS National HIV Cohort whose laboratory records were linked with very high confidence (ie, very low likelihood of linkage error), leveraging detailed information on the data linkage process (appendix pp 2–3). R version 4.0.2 was used for all statistical analyses. We followed STROBE and RECORD reporting guidelines (appendix pp 13–21).

### Role of the funding source

The funder of the study had no role in study design, data collection, data analysis, data interpretation, or writing of the report.

## Results

In 2021–22, an estimated 7·8 million people were living with HIV in South Africa, of whom 5·8 million were on ART. An estimated 5·3 million people received ART in the public sector. Between July 1, 2021, and June 30, 2022, 4 911 835 unique individuals had a viral load test in the NHLS National HIV Cohort (appendix p 22).

Our study included 3 584 203 individuals, reflecting all people living with HIV aged 15–59 years with an HIV viral load less than 200 copies per mL in the national HIV programme between July 1, 2021, and June 30, 2022 (table 1). 3 271 019 (91·3%) individuals had a baseline viral load test at a primary health clinic and 313 184 (8·7%) individuals had a baseline viral load test at a hospital. 2 588 294 (72·2%) individuals were female and 995 909 (27·8%) individuals were male. Two provinces—Gauteng and KwaZulu-Natal—accounted for over half of the cohort.

Of the 3 584 203 individuals with a baseline viral load less than 200 copies per mL, 2 858 992 (79·8%) were virally monitored at 12 months (figure; table 2; appendix pp 9–10). Individuals whose baseline viral load was their first viral load (first viral load and suppressed) were least likely to be monitored at 12 months, at 44·4% (188 868 of 425 664 individuals). Monitoring rates increased with the number of years individuals had been virally suppressed. 12-month monitoring rates were 82·0% (438 015 of 533 999 individuals) for those virally suppressed for 1 year (6–18 months) before baseline, and 91·4% (355 637 of 389 229 individuals) for those suppressed for 5 years or more before baseline. Overall, 87·2% (1 998 198 of 2 291 511) of individuals with a multiyear history of viral suppression were monitored at 12 months. Among individuals whose last viral load before baseline was 1000 copies per mL or greater, 75·0% (144 167 of 192 150 individuals) were monitored at 12 months. Viral monitoring generally increased with age and was similar for female and male individuals within each age group. All provinces had 12-month viral monitoring rates of 78·2–82·5%, except for Northern Cape at 71·5% and Gauteng at 75·7%. Rural and peri-urban areas had higher 12-month monitoring rates compared with urban areas.

Of the 2 858 992 individuals with a baseline viral load less than 200 copies per mL who were virally monitored at 12 months, 2 605 311 (91·1%) had a 12-month viral load less than 200 copies per mL (no transmission risk), 149 811 (5·2%) had a 12-month viral load of 200–999 copies per mL (almost zero transmission risk), and 103 870 (3·6%) had a 12-month viral load of 1000 copies per mL or greater, indicating elevated transmission risk (figure; appendix pp 9–10). 2755122 (96·4%) of 2858992 individuals who remained in care (with a 12-month viral load test) had a viral load less than 1000 copies per mL, indicating that 12 months after their baseline viral load less than 200 copies per mL, they still had zero or almost zero transmission risk.

Durability of viral suppression increased with history of viral suppression. For first viral load and suppressed individuals with a 12-month viral load test, 179 931 (95·3%) of 188 868 individuals had a 12-month viral load less than 1000 copies per mL. Among individuals with a previous history of viral suppression, 1 950 857 (97·6%) of 1 998 198 individuals had a 12-month viral load below 1000 copies per mL (figure; appendix pp 9–10). For those suppressed for 1 year before baseline, 422 734 (96·5%) of 438 015 individuals had a 12-month viral load less than 1000 copies per mL, whereas for those continually suppressed for at least 5 years, 350 066 (98·4%) of 355 637 individuals had a 12-month viral load less than 1000 copies per mL. By contrast, individuals with a previous viral load of 1000 copies per mL or greater (before their baseline viral load less than 200 copies per mL) had a lower likelihood of a 12-month viral load less than 1000 copies per mL, at 85·2% (122 788 of 144 167 individuals).

Among individuals monitored at 12 months, durability of viral suppression was lowest for young men and young women, and highest for women in the oldest age group. The proportion with a 12-month viral load less than 1000 copies per mL was 32 591 of 36 121 (90·2%) among male individuals aged 15–24 years, and 127 164 of 137 125 (92·7%) among female individuals aged 15–24 years. The proportion of individuals with a 12-month viral load less than 1000 copies per mL was highest among female individuals aged 45–59 years, at 599 208 (97·6%) of 614 002 individuals. There was some variability in the durability of viral suppression across provinces. The percentage of patients with a 12-month viral load less than 200 copies per mL ranged from 88·5% (151 285 of 170 947 individuals) in North West to 93·6% (175 309 of 187 307 individuals) in Western Cape. There was minimal variation across levels of urbanicity.

Viral load history was the strongest predictor of viral monitoring at 12 months (table 2; appendix p 23). The association between age and viral monitoring was attenuated after accounting for viral load history and other factors. Individuals who entered the cohort from a clinic were more likely to be monitored than those entering from a hospital (aRR 1·16, 95% CI 1·16–1·17).

A history of viral suppression was also associated with reduced risk of viral rebound (viral load ≥200 copies per mL), even after adjusting for other factors (eg, aRR 0·56, 95% CI 0·55–0·57 for 5 years suppressed *vs* first viral load; table 3; appendix p 23). Individuals with an unsuppressed viral load before cohort entry had the highest risk of viral rebound (2·53, 2·49–2·57 *vs* first viral load). After adjusting for viral load history and other demographics, viral rebound was higher among adults aged 15–24 years relative to older adults (1·37, 1·35–1·39 *vs* those aged 45–59 years) with no differences between other age groups. Women (*vs* men; 0·79, 95% CI 0·78–0·79) and individuals whose baseline viral load was at a clinic (*vs* hospital; 0·93, 0·92–0·94) had lower risk of viral rebound.

In the 2015–18 pre-dolutegravir cohort, 1 747 624 (73·3%) of 2 382 871 individuals were virally monitored at 12 months. Of those, 1 514 949 (86·7%) of 1 747 624 still had a viral load less than 200 copies per mL and 1 652 751 (94·6%) of 1 747 624 still had a viral load less than 1000 copies per mL. Between 2015–18 and 2021–23, the percentage of individuals not monitored at 12 months fell from 26·7% (635 247 of 2 382 871 individuals) to 20·2% (725 211 of 3 584 203 individuals), a 24·1% relative decline (p<0·0001); the percentage with viral load greater than 1000 copies per mL at 12 months fell from 5·4% (94 873 of 1 747 624 individuals) to 3·6% (103 870 of 2 858 992 individuals), a 33·1% relative decline (p<0·0001); and the percentage who either were not monitored or were monitored but had a viral load greater than 1000 copies per mL fell from 30·6% (730 120 of 2 382 871 individuals) to 23·1% (829 081 of 3 584 203 individuals), a 24·5% relative decline (p<0·0001; appendix pp 9–10).

Results were very similar in sensitivity analyses restricting the data to clinics, expanding the definition of viral load monitoring to 4–18 months, restricting the data to individuals with baseline viral load less than 50 copies per mL, and restricting the data to records in the NHLS National HIV Cohort that were linked with very high confidence (table 4).

## Discussion

We assessed 12-month durability of viral suppression in South Africa’s national HIV programme to guide dissemination of U=U as a prevention strategy. WHO guidelines indicate zero risk of HIV transmission with viral load less than 200 copies per mL and almost zero risk with viral load 200–999 copies per mL.^6,7^ Among individuals with a baseline viral load less than 200 copies per mL who were monitored at 12 months, 96·4% still had a viral load less than 1000 copies per mL, implying zero or almost zero transmission risk. These findings—from a national HIV cohort—should generate confidence that individuals with viral load less than 200 copies per mL can rely on U=U so long as they stay on ART.

The science of U=U is well established. However, for U=U to have practical value, clinicians and people living with HIV and their partners must have confidence that a person with a viral load less than 200 copies per mL will remain suppressed until their next viral load test (usually at 12 months), as long as they continue to take their medication. Previous studies in sub-Saharan Africa have reported 12-month rates of viral rebound varying from 1·4% to 20·2%.^13–18^ However, these studies covered narrower populations and contexts, focused on earlier periods when ART involved complex regimens^29^ with worse side-effects,^30^ and used definitions of viral rebound that did not correspond to WHO transmission risk categories. Other studies in the region did not report data needed to compute annual rates of viral rebound. Studies from Europe and North America have reported rates of viral rebound similar to our findings, although none assessed rebound relative to WHO risk categories, hindering direct comparisons. To date, most studies of viral rebound have been motivated by the goal of screening for treatment failure and drug resistance rather than assessing HIV transmission risk and implications for U=U as a prevention strategy (appendix pp 4–8).

Not all individuals in our study remained virally suppressed at 12 months. Although 96·4% of individuals with a 12-month viral load still had zero or almost zero transmission risk, 3·6% had a viral load of 1000 copies per mL or greater, signifying elevated transmission risk. These rare cases of viral rebound most likely followed adherence lapses or treatment interruptions among individuals who subsequently returned to care and had a viral load test within the 6–18-month window. Although we do not have data on treatment adherence, a Ugandan study of individuals who never missed an ART visit reported even lower (1·4%) viral rebound,^14^ suggesting durability is high for individuals without treatment interruptions. U=U guidelines clearly indicate that individuals who have treatment interruptions should not depend on U=U until they reestablish viral suppression. Per national guidelines, the 3·6% of individuals with viral loads greater than 1000 copies per mL—and the 5·2% with viral loads 200–999 copies per mL—are flagged for enhanced adherence counselling, repeat viral monitoring at 3 months, and regimen switch if necessary.

Not all individuals in our study were monitored at 12 months. The 20·2% of individuals without a 12-month viral load included those who had disengaged from care, as well as others who died, left the country, or transferred to the private sector. U=U guidance is clear: people who stop taking ART should not rely on U=U. For those still on ART but not virally monitored, our data underscore the importance of 12-monthly routine viral load testing to confirm continued suppression and identify individuals in need of support.

Our study captured important heterogeneity in patient experiences. Individuals with a history of viral suppression were more likely to be monitored and to sustain viral suppression at 12 months, consistent with previous research.^31,32^ By contrast, previous viraemia strongly predicted risk of future rebound, suggesting that some individuals might face persistent barriers to adherence that need to be addressed before relying on U=U. Previous studies show that with continued adherence and viral suppression, such individuals can reach levels of durability similar to individuals without previous viraemia.^32,33^ Low levels of viral monitoring (<50%) among new initiators are consistent with high rates of disengagement in the first year on ART,^34^ although viral suppression was durable among those retained and monitored.

Adolescents (aged 15–24 years) had lower rates of monitoring and viral suppression than older individuals, consistent with previous studies.^31,32^ Our multivariable models indicated that adolescents’ high risk of disengagement is entirely attributable to the fact that they are likely to be new to ART. Still, youth remained a risk factor for viral rebound among those who remained in care. Men were significantly more likely to have viral rebound, consistent with sex disparities across the HIV care cascade.^35^ Higher viral load monitoring and suppression at clinics (*vs* hospitals) might reflect improved continuity of care at primary health facilities. Increases in viral monitoring and suppression between 2015–18 and 2021–23 could be related to several factors, including the roll-out of dolutegravir-based treatment regimens and more widespread adoption of viral monitoring in routine clinical care.

Our analysis included all individuals with documented viral suppression in South Africa’s public sector HIV programme from July 1, 2021, to June 30, 2022. With 3·6 million individuals, to our knowledge this is the largest analysis of viral rebound to date and reflects the full diversity of South Africa’s public sector HIV programme. We followed up all individuals for 18 months—regardless of where they sought care—and obtained viral load histories, leveraging novel linkage of South Africa’s national laboratory database to create the NHLS National HIV Cohort. We aligned our analysis with WHO transmission risk thresholds so that the findings would be readily interpretable by clinicians, policy makers, and people living with HIV and their partners considering U=U as an HIV prevention strategy.

Our study has limitations. First, as U=U is advised only for adult individuals with viral load less than 200 copies per mL, our study excluded people who never reached viral suppression. Second, the NHLS cohort lacked data on ART initiation, drug regimens, clinic visits, and symptoms, which could be valuable in understanding predictors of viral rebound. Third, the 20·2% without a 12-month viral load test includes individuals who remained on ART but were not virally monitored and individuals who transferred to the private sector; thus, 79·8% should be interpreted as a lower bound on 12-month retention on ART. Fourth, our estimates of the durability of viral suppression cannot be directly applied to the 20·2% of people who were not virally monitored. People who disengaged from care might face barriers that could hamper durable viral suppression. Fifth, linkage uncertainty in the NHLS National HIV Cohort could lead to bias, as with any record linkage study. Leveraging access to the underlying linkage probabilities, we found that 86% of individual records were linked with very high confidence and that estimates were similar after restricting the analysis to these records.

In conclusion, viral suppression is highly durable among people who remain in care and on ART in South Africa’s national HIV programme. Our findings should give people with HIV and providers confidence in U=U as a tool for HIV prevention even where routine viral loads are only tested annually.

## Supplementary Material

MMC1

## Figures and Tables

**Figure:**
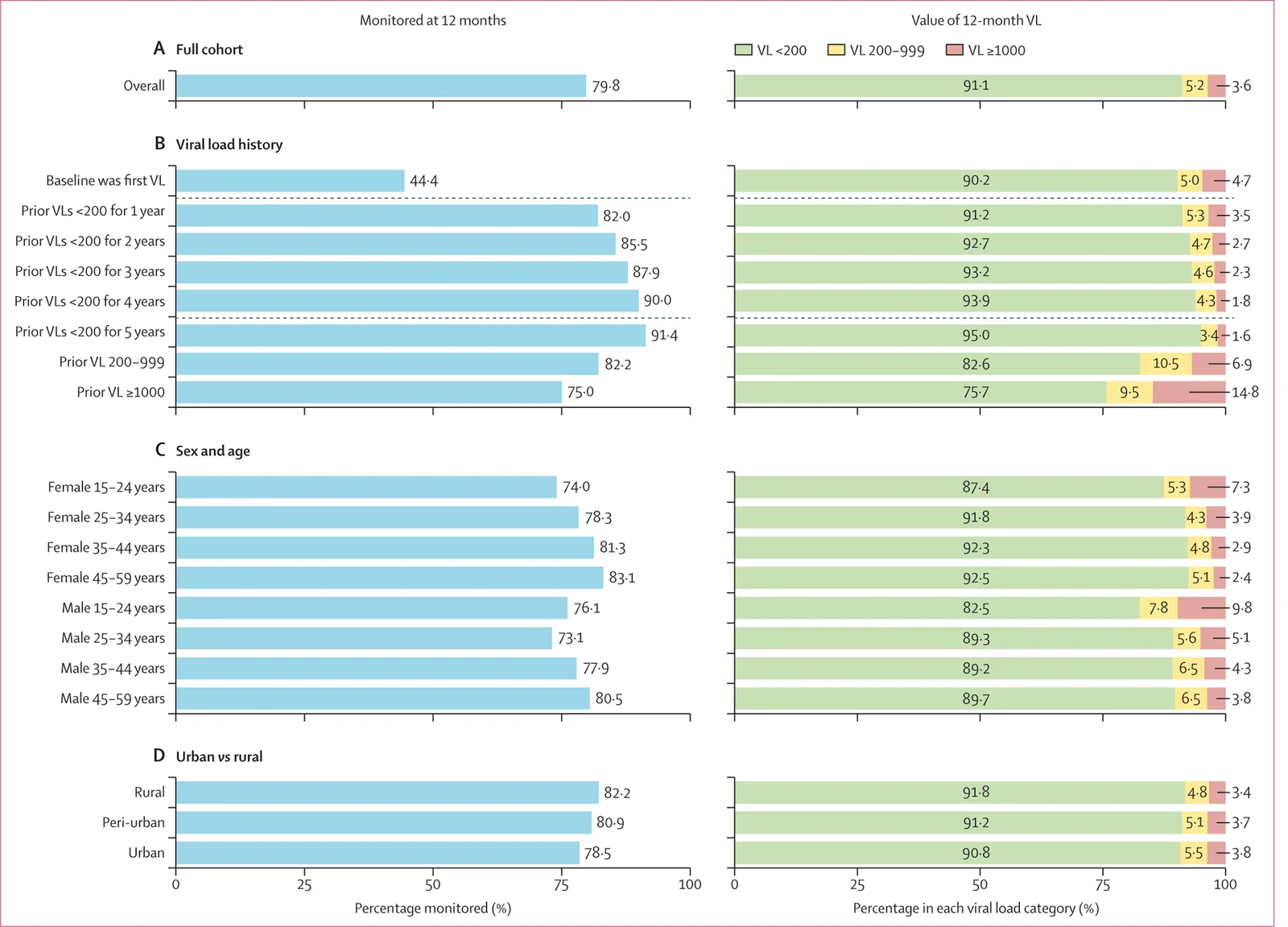
Viral load monitoring and viral load at 12 months in the full cohort (A) and stratified by viral load history (B), sex and age (C), and urban *vs* rural (D) The denominator for graphs in the right column is patients who were monitored at 12 months. Underlying data for the figure are presented in the appendix (pp 9–10). VL=viral load.

**Table 1:** Demographic characteristics of the study population

	Number of individuals (n=3 584 203)
**Sex**	
Female	2 588 294 (72·2%)
Male	995 909 (27·8%)
**Age at study entry, years**	
15–24	232 700 (6·5%)
25–34	909 972 (25·4%)
35–44	1 294 809 (36·1%)
45–59	1 146 722 (32·0%)
**Facility type at study entry**	
Clinic	3 271 019 (91·3%)
Hospital	313 184 (8·7%)
**Province at study entry**	
Eastern Cape	356 665 (10·0%)
Free State	208 932 (5·8%)
Gauteng	871 182 (24·3%)
KwaZulu-Natal	1 068 031 (29·8%)
Limpopo	233 321 (6·5%)
Mpumalanga	372 302 (10·4%)
Northern Cape	38 059 (1·1%)
North West	208 632 (5·8%)
Western Cape	227 079 (6·3%)
**Viral load history**	
First viral load and suppressed	425 664 (13·7%)
Previous viral load, continuously suppressed	
1 year	533 999 (17·2%)
2 years	423 388 (13·7%)
3 years	362 939 (11·7%)
4 years	581 956 (18·8%)
≥5 years	389 229 (12·6%)
Previous viral load, unsuppressed	
200–999 copies per mL	191 771 (6·2%)
≥1000 copies per mL	192 150 (6·2%)
**Urbanicity**	
Rural	1 057 041 (29·5%)
Peri-urban	265 409 (7·4%)
Urban	2 261 753 (63·1%)

Data are n (%). Viral load history categories do not sum to 100% because some patients did not fit into any of the categories (eg, patients who had a previous suppressed viral load, but it occurred less than 6 months before entry).

**Table 2:** Patient characteristics associated with viral monitoring at 12 months

	Percentage virally monitored	Crude RR (95%CI)	Adjusted RR (95% CI)
**Viral load history**			
First viral load and suppressed	44·4% (188 868/425 664)	1 (ref)	1 (ref)
Continuously suppressed for 1 year	82·0% (438 015/533 999)	1·85 (1·84–1·86)	1·82 (1·81–1·83)
Continuously suppressed for 2 years	85·5% (361 939/423 388)	1·93 (1·92–1·93)	1·89 (1·89–1·90)
Continuously suppressed for 3 years	87·9% (318 863/362 939)	1·98 (1·97–1·99)	1·94 (1·94–1·95)
Continuously suppressed for 4 years	90·0% (523 744/581 956)	2·03 (2·02–2·04)	1·99 (1·98–1·99)
Continuously suppressed for 5 years	91·4% (355 637/389 229)	2·06 (2·05–2·07)	2·01 (2·00–2·02)
Previous viral load and unsuppressed Last viral load 200–999 copies per mL	82·2% (157 549/191 771)	1·85 (1·84–1·86)	1·83 (1·82–1·83)
Last viral load ≥1000 copies per mL	75·0% (144 167/192 150)	1·69 (1·68–1·70)	1·68 (1·67–1·69)
**Sex**			
Male	78·1% (777 702/995 909)	1 (ref)	1 (ref)
Female	80·4% (2 081 290/2 588 294)	1·03 (1·03–1·03)	1·02 (1·01–1·02)
**Age, years**			
15–24	74·5% (173 246/232 700)	0·90 (0·89–0·90)	1·01 (1·00–1·01)
25–34	77·4% (703 992/909 972)	0·94 (0·94–0·94)	0·99 (0·99–0·99)
35–44	80·3% (1 039 501 /1 294 809)	0·98 (0·98–0·98)	0·99 (0·99–0·99)
45–59	82·2% (942 253 /1 146 722)	1 (ref)	1 (ref)
**Facility at baseline**			
Hospital	65·6% (205 324/313 184)	1 (ref)	1 (ref)
Clinic	81·1% (2 653 668/3 271 019)	1·26 (1·25–1·26)	1·16 (1·16–1·17)
**Province**			
KwaZulu-Natal	81·8% (873 637/1 068 031)	1 (ref)	1 (ref)
Eastern Cape	78·2% (278 886/356 665)	0·96 (0·96–0·96)	0·96 (0·96–0·96)
Free State	80·6% (168 419/208 932)	0·99 (0·99–0·99)	0·98 (0·98–0·98)
Gauteng	75·7% (659 669/871 182)	0·92 (0·92–0·92)	0·95 (0·95–0·95)
Limpopo	80·3% (187 319 /233 321)	0·98 (0·98–0·98)	0·98 (0·98–0·99)
Mpumalanga	82·1% (305 593/372 302)	1·00 (1·00–1·00)	1·01 (1·00–1·01)
Northern Cape	71·5% (27 215/38 059)	0·88 (0·87–0·88)	0·89 (0·88–0·89)
North West	81·9% (170 947/208 632)	1·00 (1·00–1·00)	1·01 (1·01–1·01)
Western Cape	82·5% (187 307 /227 079)	1·01 (1·01–1·02)	1·00 (1·00–1·01)

The estimation sample includes the complete study population. RR=risk ratio.

**Table 3:** Patient characteristics associated with viral rebound at 12 months

	Percentage with viral rebound	Crude RR (95% CI)	Adjusted RR (95% CI)
**Viral load history**			
First viral load and suppressed	9·8% (18 467/188 868)	1 (ref)	1 (ref)
Continuously suppressed for 1 year	8·8% (38 379/438 015)	0·90 (0·88–0·91)	0·93 (0·92–0·95)
Continuously suppressed for 2 years	7·3% (26 524/361 939)	0·75 (0·74–0·76)	0·79 (0·78–0·80)
Continuously suppressed for 3 years	6·8% (21 781/318 863)	0·70 (0·69–0·71)	0·73 (0·72–0·75)
Continuously suppressed for 4 years	6·1% (32 138/523 744)	0·63 (0·62–0·64)	0·67 (0·66–0·68)
Continuously suppressed for 5 years	5·0% (17 644/355 637)	0·51 (0·50–0·52)	0·56 (0·55–0·57)
Previous viral load and unsuppressed			
Last viral load 200–999 copies per mL	17·4% (27 415/157 549)	1·78 (1·75–1·81)	1·78 (1·75–1·81)
Last viral load ≥1000 copies per mL	24·3% (35 015/144 167)	2·48 (2·44–2·53)	2·53 (2·49–2·57)
**Sex**			
Male	10·9% (84 562/777 702)	1 (ref)	1 (ref)
Female	8·1% (169 119/2 081 290)	0·74 (0·74–0·75)	0·79 (0·78–0·79)
**Age, years**			
15–24	13·6% (23 562/173 246)	1·61 (1·59–1·64)	1·37 (1·35–1·39)
25–34	8·6% (60 816/703 992)	1·01 (1·00–1·02)	0·99 (0·98–1·00)
35–44	8·6% (89 358/1 039 501)	1·01 (1·00–1·02)	1·00 (0·99–1·01)
45–59	8·5% (79 945/942 253)	1 (ref)	1 (ref)
**Facility at baseline**			
Hospital	10·5% (21 536/205 324)	1 (ref)	1 (ref)
Clinic	8·7% (232 145/2 653 668)	0·82 (0·81–0·84)	0·93 (0·92–0·94)
**Province**			
KwaZulu-Natal	8·4% (73 087/873 637)	1 (ref)	1 (ref)
Eastern Cape	7·8% (21 722/278 886)	0·93 (0·92–0·95)	0·89 (0·88–0·90)
Free State	8·2% (13 879/168 419)	0·99 (0·97–1·01)	1·01 (0·99–1·03)
Gauteng	11·1% (73 181/659 669)	1·34 (1·32–1·35)	1·28 (1·27–1·30)
Limpopo	8·9% (16 657/187 319)	1·07 (1·05–1·09)	1·03 (1·01–1·05)
Mpumalanga	6·9% (21 062/305 593)	0·82 (0·81–0·83)	0·83 (0·81–0·84)
Northern Cape	8·9% (2 433/27 215)	1·08 (1·04–1·13)	0·95 (0·91–0·99)
North West	11·5% (19 662/170 947)	1·38 (1·36–1·41)	1·24 (1·22–1·26)
Western Cape	6·4% (11 998/187 307)	0·75 (0·73–0·76)	0·75 (0·73–0·76)

The estimation sample included all 2 858 992 members of the study population who were monitored at 12 months. Viral rebound was defined as having a 12-month viral load of at least 200 copies per mL. RR=risk ratio.

**Table 4:** Durability of viral suppression: sensitivity analyses

	Number of individuals	Percentage of individuals with any viral load at 12 months	Viral load at 12 months and associated HIV transmission risk			
			Zero risk (<200 copies per mL)	Almost zero risk (200–999 copies per mL)	Zero or almost zero risk (<1000 copies per mL)	Elevated risk (≥1000 copies per mL)
Primary analysis	3 584 203	79·8% (2 858 992/3 584 203)	91·1% (2 605 311/2 858 992)	5·2% (149 811/2 858 992)	96·4% (2 755 122/2 858 992)	3·6% (103 870/2 858 992)
Sensitivity analyses						
Baseline viral load at clinic	3 271 019	81·1% (2 653 668/3 271 019)	91·3% (2 421 523/2 653 668)	5·2% (138 908/2 653 668)	96·5% (2 560 431/2 653 668)	3·5% (93 237/2 653 668)
Viral load monitoring 4–18 months	3 584 203	81·6% (2 925 530/3 584 203)	91·0% (2 661 766/2 925 530)	5·3% (156 062/2 925 530)	96·3% (2 817 828/2 925 530)	3·7% (107 702/2 925 530)
Baseline viral load <50 copies per mL	3 146 486	80·1% (2 521 037/3 146 486)	92·3% (2 327 385/2 521 037)	4·6% (114 918/2 521 037)	96·9% (2 442 303/2 521 037)	3·1% (78 734/2 521 037)
High confidence linkages	3 072 758	77·9% (2 394 368/3 072 758)	91·5% (2 191 026/2 394 368)	5·1% (120 926/2 394 368)	96·6% (2 311 952/2 394 368)	3·4% (82 416/2 394 368)

Data include individuals with a viral load less than 200 copies per mL from July, 2021, to June, 2022, all of whom were followed up for 18 months.

## Data Availability

Access to the National Health Laboratory Service National HIV Cohort is subject to restrictions due to privacy and ethics policies set by the South African Government. Requests for access to the data can be made via a research project application to the Office of Academic Affairs and Research at the National Health Laboratory Service: https://aarms.nhls.ac.za.

## Research in context

### Evidence before this study

People living with HIV with an undetectable viral load cannot transmit HIV sexually. However, evidence is scarce on the durability of viral suppression in sub-Saharan Africa and, by extension, whether people with undetectable viral loads can rely on Undetectable=Untransmittable (U=U) as a prevention strategy in settings where viral loads are only monitored annually. We searched PubMed and Google Scholar for English-language studies with the terms “HIV” and (“Durability” OR “Persistence” OR “Viral rebound” OR “Viral suppression”) in the title, from database inception to May 12, 2025. We identified 35 cohort studies assessing viral rebound among individuals who were already virally suppressed. 14 of these studies were done in sub-Saharan Africa, of which six reported 12-month rates of viral rebound. A South African study reported 2·9% 12-month viral rebound among individuals who were stable on antiretroviral therapy (ART) and receiving differentiated models of care. A Ugandan study found 1·4% viral rebound among individuals who never missed an ART visit. Other studies reported higher rates of 12-month viral rebound: 9·4% in South Africa, 11·5% in Kenya and Uganda, 16·3% in Ghana, and 20·2% in a multicountry southern African cohort. Three of these studies reported data from the 2000s, when ART regimens were less well tolerated.

Each of the studies from sub-Saharan Africa used a different definition of viral rebound, with none aligned with WHO transmission risk categories, limiting their relevance for clinical guidance regarding U=U. Additionally, these studies were based on small, non-representative clinical cohorts; to our knowledge, no study in sub-Saharan Africa has assessed 12-month durability of viral suppression in a national HIV cohort.

### Added value of this study

This study reports 12-month durability of viral suppression in South Africa’s National Health Laboratory Service National HIV Cohort, with viral loads assessed relative to WHO transmission risk categories. People with HIV viral load less than 200 copies per mL had zero or almost zero transmission risk 12 months later, so long as they stayed on ART. This study is the first to assess durability of viral suppression in a national cohort in sub-Saharan Africa and is the largest study of viral rebound to date.

### Implications of all the available evidence

Viral suppression is highly durable among individuals who remain on ART. Low rates of viral rebound should give people living with HIV and providers confidence in recommending U=U as a tool for HIV prevention, even where routine viral loads are tested only every 12 months.
