# Supplementary material for: Durability of HIV viral suppression in South Africa: a national cohort study
Source: Lancet HIV. Author manuscript; Available in PMC 2026 Oct 5. (PMC13636457; doi:10.1016/S2352-3018(26)00079-2)
Supplement: Supplementary file 1 [file NIHMS2201905-supplement-MMC1.pdf]

THE LANCET
HIV

Supplementary appendix

This appendix formed part of the original submission and has been peer reviewed. We post it as supplied by the authors.

Supplement to: Lauren E, Bor J, Bell TG, et al. Durability of HIV viral suppression in South Africa: a national cohort study. *Lancet HIV* 2026; **13:** e548–56.

ONLINE APPENDIX

Lauren E+, Bor J+*, Gareta D, Shumba K, Muchengeti M, MacLeod W, Fox MP, Stevens W, Molapo T, Manganye M, Onoya D+, Mlisana K+. **Durability of HIV viral suppression in South Africa: a national cohort study.** *Lancet HIV.*

*Correspondence to: Jacob Bor, jbor@bu.edu
+Equal contributions

Table of contents

Methods and materials

eTable 1. Existing Literature on the Durability of Viral Suppression

eTable 2. Viral monitoring and viral suppression at 12 months: by viral load history, age and sex, and urbanicity

eTable 3. Viral monitoring and viral suppression at 12-months: by viral load history, age and sex, and urbanicity in the pre-dolutegravir era (entry to cohort between 1 March 2015 and 30 September 2016).

eTable 4. STROBE/RECORD checklist

eFigure 1. Inclusion criteria and variable definitions

eFigure 2. Relative risk of (A) viral monitoring and (B) viral rebound at 12 months

Methods and materials

Linkage of the NHLS National HIV Cohort

The National Health Laboratory Service (NHLS) conducts all VL testing for South Africa's public-sector HIV treatment program and archives all test results in a centralized database. The database includes all public sector lab results since prior to the start of the national HIV treatment programme in 2004, with the exception of lab results from KwaZulu-Natal province, which had its own laboratory system until 2010 when it joined NHLS. Data in the NHLS data base are the same data used in patient care and are not vulnerable to clerical errors that arise in manual data extraction from clinical charts.

In prior work, we created a unique person identifier within the NHLS database via a novel record linkage algorithm, enabling analysis of the NHLS database as a National HIV Cohort. The record linkage algorithm, described in detail elsewhere,[1,2] combined traditional methods for probabilistic record linkage with network analysis concepts to identify and break up clusters that were too large to be unique individuals. Linkage was conducted in collaboration with NHLS using information on first name, last name, date of birth, sex, and facility of test. After preprocessing to standardize the data fields, we searched for potential links using multiple overlapping blocking strategies. We then scored potential links using Fellegi-Sunter scoring rules for each characteristic, augmented with Jaro-Winkler scores for fuzzy string comparisons, and summed scores across the characteristics. Matches had positive scores, non-matches had negative scores. Matches on rare names (and other low probability characteristics) received higher scores than matches on common names; and non-matches received lower (negative) scores if a particular field was recorded with greater accuracy. Additional scoring rules were implemented for multiple names, nicknames, name inversions, and common date of birth errors.

Once scored, scored links between records were represented as "weighted edges" between "nodes" in a network (or graph). Discrete clusters were formed by sets of nodes (records) connected either directly or indirectly (via transitivity). These clusters were assumed to represent a single individual. Because compounding over-linkage errors can lead to implausibly large clusters, we restricted the weighted diameter of clusters to a given threshold value and eliminated the lowest scoring edges if the cluster exceeded that value. A higher threshold would return smaller clusters with lower sensitivity and higher positive predictive value; a lower threshold would return larger clusters with higher sensitivity and lower positive predictive value. At the end of this "graph-based entity resolution" step, the resulting clusters were assigned a "unique person ID".

To train and validate the record linkage algorithm, we manually coded a random sample of 1000 lab specimens and 59,000 potential matches identified for these lab specimens. Training data were used to optimize the scoring rules and weighted diameter threshold.

In validation, relative to the test data, the optimized linkage algorithm showed high accuracy with a 99% positive predictive value (1% overmatching rate) and 94% sensitivity (6% undermatching rate).

The NHLS National HIV Cohort, established via the above record linkage, includes data from January 2004 through December 2023 and allows for assessment of HIV care trajectories in the public sector, regardless of movement between facilities. For this study, we analyzed de-identified data on all HIV VLs in the NHLS National HIV Cohort. The dataset included: de-identified person identifier, sex, age, VL test date, VL test result, type of health facility that ordered the test, and province of the health facility.

Robustness to Record Linkage Errors

With access to underlying (de-identified) data on the record linkage structure of the NHLS database, we were able to identify clusters (individuals) that changed with different values of the weighted diameter threshold, and individuals that stayed the same. Those clusters that changed did so because they included one or more uncertain links. Those that did not change were comprised exclusively of "high confidence" links with very low uncertainty.

Linkage errors can bias empirical analyses of linked data, although the extent of the bias depends on the type of analysis and extent of over- and undermatching error. To assess the robustness of our main findings, we therefore replicated the analysis in the subset of the data (86% of individuals) linked with high confidence, excluding those individuals linked with greater uncertainty. We found nearly identical results in this subset of the data, indicating that record linkage errors were not a significant source of bias in our analysis.

Works Cited in Methods and Materials

1. Bor, J., MacLeod, W., Oleinik, K., Potter, J., Brennan, A. T., Candy, S., Maskew, M., Fox, M. P., Sanne, I., Stevens, W. S., & Carmona, S. (2018). Building a national HIV cohort from routine laboratory data: Probabilistic record-linkage with graphs. *BioRxiv*. https://doi.org/10.1101/450304

2. MacLeod, W. B., Bor, J., Candy, S., Maskew, M., Fox, M. P., Bulekova, K., Brennan, A. T., Potter, J., Nattey, C., Onoya, D., Mlisana, K., Stevens, W., & Carmona, S. (2022). Cohort profile: the South African National Health Laboratory Service (NHLS) National HIV Cohort. *BMJ Open*, *12*(10), e066671. https://doi.org/10.1136/bmjopen-2022-066671

eTable 1. Existing Literature on the Durability of Viral Suppression

Search methods. To assess existing evidence on the durability of viral suppression, we searched PubMed with the terms "HIV" and ("viral rebound" or “rebound”) in the title or abstract, limiting to cohort studies, from database inception to May 22, 2025. This search returned 411 studies. We also used Google Scholar to search for articles cited by or citing the few most similar studies to ours. We identified 35 relevant studies assessing viral rebound among PLHIV who were already virally suppressed, including 14 studies conducted in sub-Saharan Africa.

Study	Country	Years of data	Definition: viral suppression	Definition: viral rebound	Sample	12-mo rate of viral rebound	Other reported measures of viral rebound
Abgrall et al (2024)[1]	France	2006-2016	Two VL <50 copies/mL	VL >1000 copies/mL or 2 VL 50-999 copies/mL	37061		12% at 2 years and 19% at 5 years after ART initiation
Bekolo et al (2024)[2]	Cameroon	2002-2020	VL <1000 copies/mL	VL >1000 copies/mL	1298		23.31% (95% CI 19.24-27.77) over study period
Benoit et al (2017)[3]	Canada	2000-2012	Two VL <50 copies/mL	Two VL >200 copies/mL	5890	13% (Indigenous, 6% Caucasian), 7% (Black)	1,072 (18%) over study period
Benzie et al (2007)[4]	UK	1996-2003	VL ≤50 copies/mL	Two VL>400 copies/ mL within 6 months	10237	9.3 per 100 PY (95% CI 8.9-9.7)	
Boateng et al (2025)[5]	Ghana	2019-2021	VL <50 copies/mL	VL ≥50 copies/mL	275	16.3 per 100 PY	13.6 per 1,000 person-months (95% CI: 10.5-17.7)
Castelnuovo et al (2016)[6]	Uganda	2004-2015	VL <400 copies/mL	Two VL>1000 copies/mL or VL >5000 copies/mL	439		74/439 (17%) over the 10-year follow-up period
Childs et al (2015)[7]	UK, Ireland	1996-2013	VL <400 copies/mL	Two VL >400 copies/mL within 6 months	900 children <18 years		170 (19%) over study period
Craw et al (2020)[8]	USA	2015-2018	VL <200 copies/mL	VL ≥200 copies/ml	6767		7.5% (95% CI: 6.7-8.4%) over study period
Crawford (2014)[9]	USA	2003-2009	VL ≤400 copies/mL	VL >400 copies/mL	658	172 (26%)	
Dhairyawan et al (2021)[10]	UK	2000-2017	VL <50 copies/mL	Two VL >50 copies/mL	6698		1,489 (22.2%) over study period

Henri et al (2023)[11]	Central African Rep.	2023	VL <1000 copies/mL	VL >1000 copies/mL	458		78/458 (17%) over study period
Kahabuka et al (2024)[12]	Tanzania	2016-2020	VL <1000 copies/mL	VL ≥1000 copies/mL	310		40% (124/310; 95% CI: 34.5-45.7) over study period
Ladak et al (2019)[13]	Canada	1996-2013	Two VL <50 copies/mL	Two VL ≥1000 copies/mL	564 people with drug use		198 (35.1%) over median follow-up time of 34.2 mo
Liu et al (2023)[14]	USA	2010-2016	2 VLs <200 copies/mL	VL ≥200 copies/mL	3496		351 (10%) over study period (2 years of follow-up)
Maina et al (2020)[15]	Kenya	2003-2019	VL ≤40 copies/mL	VL >40 copies/mL	549		49% over study period
Malagnino et al (2024)[16]	Italy	1998-2021	VL ≤50 copies/mL	Two VL >50 copies/mL	5657		654 (11.5%) over study period
Milloy et al (2012)[17]	Canada	1996-2009	VL <50 copies/mL (VL<500 pre-1999)	Two VL >1000 copies/mL	277 people with injecting drug use	12.6% (95% CI: 10.5 to 15.0)	125 (45.1%) over study period
Min et al (2020)[18]	USA	2015-2019	VL <200 copies/mL	VL ≥200 copies/mL	1242	5.1%	
Morozova et al (2025)[19]	Ukraine	2017-2018	VL <200 copies/mL	VL ≥200 copies/mL	18160	1.3 per 100 PY in ART-experienced cohort	6.87 per 100 PY in ART-naïve patients
Mujugira et al (2016)[20]	Kenya, Uganda	2008-2012	VL <80 copies/mL	VL >200 copies/mL	867 PLHIV in mixed couples	11.5%	8.8% over study period
Nachega et al (2009)[21]	Southern Africa	1999-2006	VL ≤400 copies/mL	VL >400 copies/mL	3805 adults in 9 countries	20.2%	
O'Connor et al (2017)[22]	UK	1998-2014	VL <50 copies/mL	VL >200 copies/mL or no ART use for ≥1 month	16101	7.8 per 100 PY (95% CI: 7.6-8.0)	12.6 per 100 PY in year 1 after ART start; 2.5 in years 10-15
Opoku et al (2022)[23]	Ghana	2016-2020	VL ≤50 copies/mL	VL >50 copies/mL	548		115/548 (21.0%) over five-year period
Palmer et al (2018)[24]	Canada	2000-2014	Two VL <50 copies/mL	Two VL >50 copies/mL	7,140 >29 years; 1,218 ≤29 years	9% (95% CI 8-9%) for PLHIV >29 years	11% (95% CI 10-13%) for PLHIV ≤29

Phillips et al (2004)[25]	Europe	1994-?	VL <50 copies/mL	VL >400 copies/mL	2120	4.9 per 100 PY (95% CI 4.0–5.8) for NRTI-naïve	8.0/100 PY (95% CI 7.0–9.0) for NRTI-experienced
Robertson et al (2015)[26]	USA	2005-2009	VL ≤400 copies/mL	VL >1000 copies/mL	9298		34% over study period
Rosen et al (2024)[27]	Uganda	2015-2020	VL <200 copies/mL	VL >200 copies/mL	3,101 with no missed ART visits	1.4 per 100 PY (95% CI: 1.1-1.8)	3.1 per 100 PY for those with facility switching
Sanne et al (2009)[28]	South Africa	2004-2007	VL <400 copies/ mL within 6mo of ART	Two VL >400 copies/mL, or 1 VL >5000 copies/mL	4779	9.4% (95% CI 8.5-10.3%)	16.8% at 2 years, and 20.6% at 3 years of suppression
Shigayeva et al (2022)[29]	South Africa	2012-2018	VL <400 copies/mL	VL ≥1000 copies/mL	6113 stable ART patients in DMOC	2.9 per 100 PY	
Smith et al (2009)[30]	UK, Germany	1991-2006	VL <50 copies/mL	VL >400 copies/mL	4,007 people with no prior trt failure	9.2 per 100 PY (95% CI 8.0–10.5)	
Tadesse et al (2019)[31]	Ethiopia	2017-2019	VL <150 copies/mL	VL ≥150 copies/mL	94 children 3-12 years		36 (38.3%) over study period (48 weeks of follow-up)
Tanner et al (2016)[32]	Canada	2000-2012	Two VL <50 copies/mL	Two VL >200 copies/mL	2448 MSM		295 (12%) over study period
Teasdale et al (2018)[33]	South Africa	2012-2014	VL <1000 copies/mL	VL >1000 copies/mL	234 children <12 years		47 (20.1%) over study period
Xiao et al (2025)[34]	USA	2005-2021	Two VL ≤200 copies/mL	VL >200 copies/mL			1175 (14.4%) over study period
Young et al (2015)[35]	Switzerland	1998?-2014	Two VL <50 copies/mL	Two VL ≥50 copies/mL or VL ≥1000 copies/mL	4094	5.6 per 100 PY	

Works cited in eTable 1:

1 Abgrall S, Selinger-Leneman H, Lanoy E, *et al.* Viral rebound on antiretroviral therapy in France according to region of origin, sex, and HIV acquisition group. Results from the French Hospital Database on HIV (ANRS CO4-FHDH). *HIV Med* 2024; 26: 252–64.

2 Bekolo CE, Ndeso SA, Moifo LL, *et al.* Changes in access to viral load testing, incidence rates of viral load suppression and rebound following the introduction of the 'universal test and treat' guidelines in Cameroon: A retrospective follow-up analysis. *PLOS Global Public Health* 2024; 4: e0003042.

3 Benoit AC, Younger J, Beaver K, *et al.* A comparison of virological suppression and rebound between Indigenous and non-Indigenous persons initiating combination antiretroviral therapy in a multisite cohort of individuals living with HIV in Canada. *Antivir Ther* 2017; 22: 325–35.

4 Benzie AA, Bansi LK, Sabin CA, *et al.* Increased duration of viral suppression is associated with lower viral rebound rates in patients with previous treatment failures. *AIDS* 2007; 21: 1423–30.

5 Boateng AT, Aboagye JO, Lamptey H, *et al.* Factors affecting viral suppression or rebound in people living with HIV and receiving antiretroviral therapy in Ghana. *Front Public Health* 2025; 13: 1508793.

6 Castelnuovo B, Kiragga A, Mubiru F, Kambugu A, Kamya M, Reynolds SJ. First-line antiretroviral therapy durability in a 10-year cohort of naïve adults started on treatment in Uganda. *J Int AIDS Soc* 2016; 19: 20773.

7 Childs T, Shingadia D, Goodall R, *et al.* Outcomes after viral load rebound on first-line antiretroviral treatment in children with HIV in the UK and Ireland: An observational cohort study. *Lancet HIV* 2015; 2: e151–8.

8 Craw JA, Beer L, Tie Y, Jaenicke T, Shouse RL, Prejean J. Viral Rebound Among Persons With Diagnosed HIV Who Achieved Viral Suppression, United States. *J Acquir Immune Defic Syndr (1988)* 2020; 84: 133–40.

9 Crawford TN. Poor retention in care one-year after viral suppression: A significant predictor of viral rebound. *AIDS Care - Psychological and Socio-Medical Aspects of AIDS/HIV* 2014; 26: 1393–9.

10 Dhairyawan R, Okhai H, Hill T, Sabin CA. Differences in HIV clinical outcomes amongst heterosexuals in the United Kingdom by ethnicity. *AIDS* 2021; 35: 1813.

11 Henri DSC, Dieu LJ de, Honore WS, Herman D-WR, Emmanuel F, Gresenguet G. Factors associated with viral suppression and rebound in adult HIV patients on antiretroviral therapy. *J Public Health Epidemiol* 2023; 15: 297–303.

12 Kahabuka MS, Woldeamanuel Y, Mbelele PM, *et al.* HIV viral suppression and risk of viral rebound in patients on antiretroviral therapy: a two- year retrospective cohort study in Northern Tanzania. *BMC Infect Dis* 2024; 24: 1–11.

13 Ladak F, Socias E, Nolan S, *et al.* Substance use patterns and HIV-1 RNA viral load rebound among HIV-positive illicit drug users in a Canadian setting. *Antivir Ther* 2019; 24: 19–25.

14 Liu T, Chambers LC, Hansen B, *et al.* Risk of HIV Viral Rebound in the Era of Universal Treatment in a Multicenter Sample of Persons With HIV in Primary Care. *Open Forum Infect Dis* 2023; 10: ofad257.

15 Maina EK, Mureithi H, Adan AA, Muriuki J, Lwembe RM, Bukusi EA. Incidences and factors associated with viral suppression or rebound among HIV patients on combination antiretroviral therapy from three counties in Kenya. *International Journal of Infectious Diseases* 2020; 97: 151–8.

16 Malagnino V, Cozzi-Lepri A, Svicher V, *et al.* Association between markers of hepatitis B virus infection and risk of virological rebound in people with HIV receiving antiretroviral therapy. *HIV Med* 2024; 25: 1101–11.

17 Milloy MJ, Kerr T, Buxton J, *et al.* Social and environmental predictors of plasma HIV RNA rebound among injection drug users treated with antiretroviral therapy. *J Acquir Immune Defic Syndr (1988)* 2012; 59: 393–9.

18 Min S, Gillani FS, Aung S, Garland JM, Beckwith CG. Evaluating HIV Viral Rebound Among Persons on Suppressive Antiretroviral Treatment in the Era of "Undetectable Equals Untransmittable (U = U)". *Open Forum Infect Dis* 2020; 7. DOI:10.1093/OFID/OFAA529.
19 Morozova O, Conroy K, Riabokon S, *et al.* HIV Viral Rebound on Dolutegravir, Lopinavir and Efavirenz: National Program Data Analysis from Ukraine. *J Acquir Immune Defic Syndr* 2025. DOI:10.1097/QAI.0000000000003676,.
20 Mujugira A, Celum C, Tappero JW, Ronald A, Mugo N, Baeten JM. Younger Age Predicts Failure to Achieve Viral Suppression and Virologic Rebound among HIV-1-Infected Persons in Serodiscordant Partnerships. *AIDS Res Hum Retroviruses* 2016; 32: 148–54.
21 Nachega JB, Hislop M, Nguyen H, *et al.* Antiretroviral therapy adherence, virologic and immunologic outcomes in adolescents compared with adults in southern Africa. *J Acquir Immune Defic Syndr* 2009; 51: 65–71.
22 O'Connor J, Smith C, Lampe FC, *et al.* Durability of viral suppression with first-line antiretroviral therapy in patients with HIV in the UK: an observational cohort study. *Lancet HIV* 2017; 4: e295–302.
23 Opoku S, Sakyi SA, Ayisi-Boateng NK, *et al.* Factors associated with viral suppression and rebound among adult HIV patients on treatment: a retrospective study in Ghana. *AIDS Res Ther* 2022; 19: 1–10.
24 Palmer A, Gabler K, Rachlis B, *et al.* Viral suppression and viral rebound among young adults living with HIV in Canada. *Medicine* 2018; 97: e10562.
25 Phillips AN, Ledergerber B, Horban A, *et al.* Rate of viral rebound according to specific drugs in the regimen in 2120 patients with HIV suppression. *AIDS* 2004; 18: 1795–804.
26 Robertson M, Laraque F, Mavronicolas H, Braunstein S, Torian L. Linkage and retention in care and the time to HIV viral suppression and viral rebound - New York City. *AIDS Care - Psychological and Socio-Medical Aspects of AIDS/HIV* 2015; 27: 260–7.
27 Rosen JG, Ndyanabo A, Nakawooya H, *et al.* Incidence of Health Facility Switching and Associations With HIV Viral Rebound Among Persons on Antiretroviral Therapy in Uganda: A Population-based Study. *Clinical Infectious Diseases* 2024; 78: 1591–600.
28 Sanne IM, Westreich D, Macphail AP, Rubel D, Majuba P, Van Rie A. Long term outcomes of antiretroviral therapy in a large HIV/AIDS care clinic in urban South Africa: a prospective cohort study. *J Int AIDS Soc* 2009; 12: 38.
29 Shigayeva A, Gcwensa N, Ndlovu CD, *et al.* Retention on ART and viral suppression among patients in alternative models of differentiated HIV service delivery in KwaZulu-Natal, South Africa. *PLOS Global Public Health* 2022; 2: e0000336.
30 Smith CJ, Phillips AN, Dauer B, *et al.* Factors associated with viral rebound among highly treatment-experienced HIV-positive patients who have achieved viral suppression. *HIV Med* 2009; 10: 19–27.
31 Tadesse BT, Chala A, Mukonzo J, *et al.* Rates and Correlates of Short Term Virologic Response among Treatment-Naïve HIV-Infected Children Initiating Antiretroviral Therapy in Ethiopia: A Multi-Center Prospective Cohort Study. *Pathogens* 2019; 8: 161.
32 Tanner Z, Lachowsky N, Ding E, *et al.* Predictors of viral suppression and rebound among HIV-positive men who have sex with men in a large multi-site Canadian cohort. *BMC Infect Dis* 2016; 16. DOI:10.1186/S12879-016-1926-Z.
33 Teasdale CA, Sogaula N, Yuengling KA, *et al.* HIV viral suppression and longevity among a cohort of children initiating antiretroviral therapy in Eastern Cape, South Africa. *J Int AIDS Soc* 2018; 21: e25168.
34 Xiao J, Yang X, Ma Y, *et al.* Longitudinal assessments of viral rebound among people with HIV in South Carolina: a population-based cohort study. *AIDS Care* 2025; published online Jan 2. DOI:10.1080/09540121.2024.2411270.
35 Young J, Rickenbach M, Calmy A, *et al.* Transient detectable viremia and the risk of viral rebound in patients from the Swiss HIV Cohort Study. *BMC Infect Dis* 2015; 15: 382.

eTable 2. Viral monitoring and viral suppression at 12 months: by viral load history, age and sex, and urbanicity

Panel A. Overall and stratified by viral load history

Category		N at entry to cohort	N with any VL at 12 months (N monitored)	Transmission risk at 12 months (copies/ml)			
				Zero risk	Almost zero risk	Zero or almost zero risk	Elevated risk
				(<200)	(200-999)	(<1000)	(≥1000)
**Overall**		3,584,203	2,858,992 (79.8%)	2,605,311 (91.1%)	149,811 (5.2%)	2,755,122 (96.4%)	103,870 (3.6%)
**Baseline VL<200 was first VL**		425,664	188,868 (44.4%)	170,401 (90.2%)	9,530 (5.0%)	179,931 (95.3%)	8,937 (4.7%)
**Had previous VLs<200 cp/ml and was virally suppressed for X years before baseline**	**≥1 year**	2,291,511	1,998,198 (87.2%)	1,861,732 (93.2%)	89,125 (4.5%)	1,950,857 (97.6%)	47,341 (2.4%)
	**1 year**	533,999	438,015 (82.0%)	399,636 (91.2%)	23,098 (5.3%)	422,734 (96.5%)	15,281 (3.5%)
	**2 years**	423,388	361,939 (85.5%)	335,415 (92.7%)	16,900 (4.7%)	352,315 (97.3%)	9,624 (2.7%)
	**3 years**	362,939	318,863 (87.9%)	297,082 (93.2%)	14,570 (4.6%)	311,652 (97.7%)	7,211 (2.3%)
	**4 years**	581,956	523,744 (90.0%)	491,606 (93.9%)	22,484 (4.3%)	514,090 (98.2%)	9,654 (1.8%)
	**≥5 years**	389,229	355,637 (91.4%)	337,993 (95.0%)	12,073 (3.4%)	350,066 (98.4%)	5,571 (1.6%)
**Had previous VL≥200 cp/ml**	**200-999 cp/ml**	191,771	157,549 (82.2%)	130,134 (82.6%)	16,589 (10.5%)	146,723 (93.1%)	10,826 (6.9%)
	**≥1000 cp/ml**	192,150	144,167 (75.0%)	109,152 (75.7%)	13,636 (9.5%)	122,788 (85.2%)	21,379 (14.8%)

Note: see Table 1 note for viral load history definitions.

Panel B. Stratified by sex and age

Sex	Age	N at entry to cohort	N with any VL at 12 months (N monitored)	Transmission risk at 12 months (copies/ml)			
				Zero risk	Almost zero risk	Zero or almost zero risk	Elevated risk
				(<200)	(200-999)	(<1000)	(≥1000)
**Female**	**15-24**	185,258	137,125 (74.0%)	119,895 (87.4%)	7,269 (5.3%)	127,164 (92.7%)	9,961 (7.3%)
	**25-34**	748,322	585,835 (78.3%)	537,618 (91.8%)	25,352 (4.3%)	562,970 (96.1%)	22,865 (3.9%)
	**35-44**	915,853	744,328 (81.3%)	686,808 (92.3%)	36,005 (4.8%)	722,813 (97.1%)	21,515 (2.9%)
	**45-59**	738,861	614,002 (83.1%)	567,850 (92.5%)	31,358 (5.1%)	599,208 (97.6%)	14,794 (2.4%)
**Male**	**15-24**	47,442	36,121 (76.1%)	29,789 (82.5%)	2,802 (7.8%)	32,591 (90.2%)	3,530 (9.8%)
	**25-34**	161,650	118,157 (73.1%)	105,558 (89.3%)	6,578 (5.6%)	112,136 (94.9%)	6,021 (5.1%)
	**35-44**	378,956	295,173 (77.9%)	263,335 (89.2%)	19,084 (6.5%)	282,419 (95.7%)	12,754 (4.3%)
	**45-59**	407,861	328,251 (80.5%)	294,458 (89.7%)	21,363 (6.5%)	315,821 (96.2%)	12,430 (3.8%)

Panel C. Stratified by urbanicity

Urbanicity	N at entry to cohort	N with any VL at 12 months (N monitored)	Transmission risk at 12 months (copies/ml)			
			Zero risk	Almost zero risk	Zero or almost zero risk	Elevated risk
			(<200)	(200-999)	(<1000)	(≥1000)
**Rural**	1,057,041	869,353 (82.2%)	798,273 (91.8%)	41,759 (4.8%)	840,032 (96.6%)	29,321 (3.4%)
**Peri-Urban**	265,409	214,604 (80.9%)	195,650 (91.2%)	11,038 (5.1%)	206,688 (96.3%)	7,916 (3.7%)
**Urban**	2,261,753	1,775,035 (78.5%)	1,611,388 (90.8%)	97,014 (5.5%)	1,708,402 (96.2%)	66,633 (3.8%)

eTable 3. Viral monitoring and viral suppression at 12 months: by viral load history, age and sex, and urbanicity in the pre-dolutegravir cohort (entry to cohort between 1 March 2015 and 30 September 2016).

Panel A. Overall and stratified by viral load history

Category		N at entry to cohort	N with any VL at 12 months (N monitored)	Transmission risk at 12 months (copies/ml)			
				Zero risk	Almost zero risk	Zero or almost zero risk	Elevated risk
				(<200)	(200-999)	(<1000)	(≥1000)
**Overall**		2,382,871	1,747,624 (73.3%)	1,514,949 (86.7%)	137,802 (7.9%)	1,652,751 (94.6%)	94,873 (5.4%)
**Baseline VL<200 was first VL**		788,737	476,951 (60.5%)	420,445 (88.2%)	32,548 (6.8%)	452,993 (95.0%)	23,958 (5.0%)
**Had previous VLs<200 cp/ml and was virally suppressed for X years before baseline**	**≥1 year**	863,511	716,483 (83.0%)	643,271 (89.8%)	49,277 (6.9%)	692,548 (96.7%)	23,935 (3.3%)
	**1 year**	416,139	335,538 (80.6%)	292,198 (87.1%)	28,767 (8.6%)	320,965 (95.7%)	14,573 (4.3%)
	**2 years**	238,457	200,146 (83.9%)	181,389 (90.6%)	12,935 (6.5%)	194,324 (97.1%)	5,822 (2.9%)
	**3 years**	127,878	109,860 (85.9%)	102,251 (93.1%)	5,210 (4.7%)	107,461 (97.8%)	2,399 (2.2%)
	**4 years**	50,083	43,476 (86.8%)	41,286 (95.0%)	1,434 (3.3%)	42,720 (98.3%)	756 (1.7%)
	**≥5 years**	30,954	27,463 (88.7%)	26,147 (95.2%)	931 (3.4%)	27,078 (98.6%)	385 (1.4%)
**Had previous VL≥200 cp/ml**	**200-999 cp/ml**	219,404	171,915 (78.4%)	135,645 (78.9%)	22,474 (13.1%)	158,119 (92.0%)	13,796 (8.0%)
	**≥1000 cp/ml**	224,324	165,106 (73.6%)	124,602 (75.5%)	17,384 (10.5%)	141,986 (86.0%)	23,120 (14.0%)

Note: see Table 1 note for viral load history definitions.

Panel B. Stratified by sex and age

Sex	Age	N at entry to cohort	N with any VL at 12 months (N monitored)	Transmission risk at 12 months (copies/ml)			
				Zero risk	Almost zero risk	Zero or almost zero risk	Elevated risk
				(<200)	(200-999)	(<1000)	(≥1000)
**Female**	**15-24**	169,598	111,068 (65.5%)	95,446 (85.9%)	7,139 (6.4%)	102,585 (92.4%)	8,483 (7.6%)
	**25-34**	611,861	444,318 (72.6%)	389,353 (87.6%)	31,676 (7.1%)	421,029 (94.8%)	23,289 (5.2%)
	**35-44**	568,493	432,748 (76.1%)	379,160 (87.6%)	33,988 (7.9%)	413,148 (95.5%)	19,600 (4.5%)
	**45-59**	377,078	288,217 (76.4%)	253,294 (87.9%)	23,361 (8.1%)	276,655 (96.0%)	11,562 (4.0%)
**Male**	**15-24**	26,414	17,740 (67.2%)	14,093 (79.4%)	1,538 (8.7%)	15,631 (88.1%)	2,109 (11.9%)
	**25-34**	153,268	105,246 (68.7%)	89,633 (85.2%)	8,305 (7.9%)	97,938 (93.1%)	7,308 (6.9%)
	**35-44**	263,418	191,792 (72.8%)	161,922 (84.4%)	17,133 (8.9%)	179,055 (93.4%)	12,737 (6.6%)
	**45-59**	212,741	156,495 (73.6%)	132,048 (84.4%)	14,662 (9.4%)	146,710 (93.7%)	9,785 (6.3%)

Panel C. Stratified by urbanicity

Urbanicity	N at entry to cohort	N with any VL at 12 months (N monitored)	Transmission risk at 12 months (copies/ml)			
			Zero risk	Almost zero risk	Zero or almost zero risk	Elevated risk
			(<200)	(200-999)	(<1000)	(≥1000)
**Rural**	726,499	536,691 (73.9%)	477,339 (88.9%)	33,840 (6.3%)	511,179 (95.2%)	25,512 (4.8%)
**Peri-Urban**	185,032	135,219 (73.1%)	121,762 (90.0%)	7,179 (5.3%)	128,941 (95.4%)	6,278 (4.6%)
**Urban**	1,471,340	1,075,714 (73.1%)	915,848 (85.1%)	96,783 (9.0%)	1,012,631 (94.1%)	63,083 (5.9%)

eTable 4. The RECORD statement – checklist of items, extended from the STROBE statement, that should be reported in observational studies using routinely collected health data

	Item No.	STROBE items	Location in manuscript where items are reported	RECORD items	Location in manuscript where items are reported
**Title and abstract**					
	1	(a) Indicate the study's design with a commonly used term in the title or the abstract (b) Provide in the abstract an informative and balanced summary of what was done and what was found	(a) Abstract (methods) (page 2)<br><br>(b) Abstract (Findings and interpretation) (page 2)	RECORD 1.1: The type of data used should be specified in the title or abstract. When possible, the name of the databases used should be included.<br><br>RECORD 1.2: If applicable, the geographic region and timeframe within which the study took place should be reported in the title or abstract.<br><br>RECORD 1.3: If linkage between databases was conducted for the study, this should be clearly stated in the title or abstract.	Abstract (methods) (page 2)<br><br>Abstract (methods) (page 2)<br><br>Abstract (methods) (page 2)
**Introduction**					
Background rationale	2	Explain the scientific background and rationale for the investigation being reported	Paragraphs 1-3 (page 4)		
Objectives	3	State specific objectives, including any prespecified hypotheses	Paragraph 4 (page 4)		
**Methods**					
Study Design	4	Present key elements of study design early in the paper	Reported in the following sections: Data source, study		

			population, outcome measures (page 5, 6)		
Setting	5	Describe the setting, locations, and relevant dates, including periods of recruitment, exposure, follow-up, and data collection	Reported in study setting, data sources, study population (page 4, 5)		
Participants	6	*(a)* *Cohort study* - Give the eligibility criteria, and the sources and methods of selection of participants. Describe methods of follow-up<br>*Case-control study* - Give the eligibility criteria, and the sources and methods of case ascertainment and control selection. Give the rationale for the choice of cases and controls<br>*Cross-sectional study* - Give the eligibility criteria, and the sources and methods of selection of participants<br><br>*(b)* *Cohort study* - For matched studies, give matching criteria and number of exposed and unexposed<br>*Case-control study* - For matched studies, give matching criteria and the number of controls per case	(a) Reported in data sources, study population, outcome measures (page 5, 6)<br><br>(b) N/A (not a matched study)	RECORD 6.1: The methods of study population selection (such as codes or algorithms used to identify subjects) should be listed in detail. If this is not possible, an explanation should be provided.<br><br>RECORD 6.2: Any validation studies of the codes or algorithms used to select the population should be referenced. If validation was conducted for this study and not published elsewhere, detailed methods and results should be provided.<br><br>RECORD 6.3: If the study involved linkage of databases, consider use of a flow diagram or other graphical display to demonstrate the data linkage process, including the number of individuals with linked data at each stage.	Reported in Data sources (page 5)<br><br>Reported in Data sources (page 5)<br><br>Flow diagram of inclusion criteria provided in the appendix (eFigure 1).<br><br>Referenced cohort profiles demonstrating the data linkage process in the Data source section (page 5).

					Explanation on the linkage process is also provided in the appendix (under methods and materials, “Linkage of the NHLS National HIV Cohort”, “Robustness to Record Linkage errors”)
Variables	7	Clearly define all outcomes, exposures, predictors, potential confounders, and effect modifiers. Give diagnostic criteria, if applicable.	Reported in Outcomes measures and Analyses (page 5, 6)	RECORD 7.1: A complete list of codes and algorithms used to classify exposures, outcomes, confounders, and effect modifiers should be provided. If these cannot be reported, an explanation should be provided.	Reported in Data source, outcome measures (page 5,6), Appendix “Methods and materials”

Data sources/ measurement	8	For each variable of interest, give sources of data and details of methods of assessment (measurement).<br>Describe comparability of assessment methods if there is more than one group	Reported in Data source (page 5)		

Bias	9	Describe any efforts to address potential sources of bias	Reported in outcome measures, analyses section (page 5, 6)		
Study size	10	Explain how the study size was arrived at	Reported in Study population (page 5), paragraph 1 of results section (page 7), and Appendix (eFigure 1)		
Quantitative variables	11	Explain how quantitative variables were handled in the analyses. If applicable, describe which groupings were chosen, and why	Reported in outcome measures, analyses section (page 5, 6)		

Statistical methods	12	(a) Describe all statistical methods, including those used to control for confounding (b) Describe any methods used to examine subgroups and interactions<br>(c) Explain how missing data were addressed<br>(d) *Cohort study* - If applicable, explain how loss to follow-up was addressed<br>*Case-control study* - If applicable, explain how matching of cases and controls was addressed<br>*Cross-sectional study* - If applicable, describe analytical methods taking account of sampling strategy<br>(e) Describe any sensitivity analyses	(a) Reported in outcome measures, analyses section (page 5, 6)<br>(b) Reported in outcome measures, analyses section (page 5, 6)<br>(c) Limitations section (page 10)<br>(d) Limitations section (page 10)<br>(e) Paragraph 3 of analyses section (page 6)		
Data access and cleaning methods		..		RECORD 12.1: Authors should describe the extent to which the investigators had access to the database population used to create the study population.	Ethical consideration section (page 7)
				RECORD 12.2: Authors should provide information on the data cleaning methods used in the study.	See cohort profile referenced in the data source section (page 5), appendix (under methods and

					materials, “Linkage of the NHLS National HIV Cohort”, “Robustness to Record Linkage errors”)
Linkage		..		RECORD 12.3: State whether the study included person-level, institutional-level, or other data linkage across two or more databases. The methods of linkage and methods of linkage quality evaluation should be provided.	Reported in Data source (page 5), appendix (under methods and materials, “Linkage of the NHLS National HIV Cohort”, “Robustness to Record Linkage errors”)
**Results**					
Participants	13	(a) Report the numbers of individuals at each stage of the study (*e.g.*, numbers potentially eligible, examined for eligibility, confirmed eligible, included in the study, completing follow-up, and analysed)<br>(b) Give reasons for nonparticipation at each stage.<br>(c) Consider use of a flow diagram	(a, b, c) Results section (page 7, 8), Appendix eFigure 1	RECORD 13.1: Describe in detail the selection of the persons included in the study (*i.e.,* study population selection) including filtering based on data quality, data availability and linkage. The selection of included persons can be described in the text and/or by means of the study flow diagram.	Appendix eFigure1, cohort profile cited in the “data source” section (page 5)

Descriptive data	14	(a) Give characteristics of study participants (*e.g.*, demographic, clinical, social) and information on exposures and potential confounders<br>(b) Indicate the number of participants with missing data for each variable of interest (c) *Cohort study* - summarise follow-up time (*e.g.*, average and total amount)	(a) Table 1, Resuls section (page 7, 8)<br>(b) Results section (page 7, 8)<br>(c) Results section (page 7, 8)		
Outcome data	15	*Cohort study* - Report numbers of outcome events or summary measures over time<br>*Case-control study* - Report numbers in each exposure	Results section (page 7, 8), Figure 1, table 2-4		
		category, or summary measures of exposure<br>*Cross-sectional study* - Report numbers of outcome events or summary measures			

Main results	16	(a) Give unadjusted estimates and, if applicable, confounderadjusted estimates and their precision (e.g., 95% confidence interval). Make clear which confounders were adjusted for and why they were included (b) Report category boundaries when continuous variables were categorized<br>(c) If relevant, consider translating estimates of relative risk into absolute risk for a meaningful time period	(a-c) Results section (page 7, 8), Figure 1, table 2-4		
Other analyses	17	Report other analyses done—e.g., analyses of subgroups and interactions, and sensitivity analyses	Results section (page 11, 12), Figure 1-2 (page 21), Table 1-3 (page 21, 22)		
**Discussion**					
Key results	18	Summarise key results with reference to study objectives	Paragraph 1 of discussion (page 9)		
Limitations	19	Discuss limitations of the study, taking into account sources of potential bias or imprecision. Discuss both direction and magnitude of any potential bias	Paragraph 8 of discussion (limitations) (page 10)	RECORD 19.1: Discuss the implications of using data that were not created or collected to answer the specific research question(s). Include discussion of misclassification bias, unmeasured confounding, missing data, and changing eligibility over time, as they pertain to the study being reported.	Paragraph 8 of discussion (limitations) (page 10)

Interpretation	20	Give a cautious overall interpretation of results considering objectives, limitations, multiplicity of analyses, results from similar studies, and other relevant evidence	Paragraph 1-6 of discussion (page 9,10)		
Generalisability	21	Discuss the generalisability (external validity) of the study results	Paragraph 2-5 of discussion (page 9,10)		
**Other Information**					
Funding	22	Give the source of funding and the role of the funders for the present study and, if applicable, for the original study on which the present article is based	Disclosed in role of funding source (page 7), acknowledgements (page 11)		
Accessibility of protocol, raw data, and programming code		..		RECORD 22.1: Authors should provide information on how to access any supplemental information such as the study protocol, raw data, or programming code.	Disclosed in data sharing statement (page 10-11)

*Reference: Benchimol EI, Smeeth L, Guttmann A, Harron K, Moher D, Petersen I, Sørensen HT, von Elm E, Langan SM, the RECORD Working Committee. The REporting of studies Conducted using Observational Routinely-collected health Data (RECORD) Statement. *PLoS Medicine* 2015; in press.

*Checklist is protected under Creative Commons Attribution (CC BY) license.

eFigure 1. Inclusion criteria and variable definitions

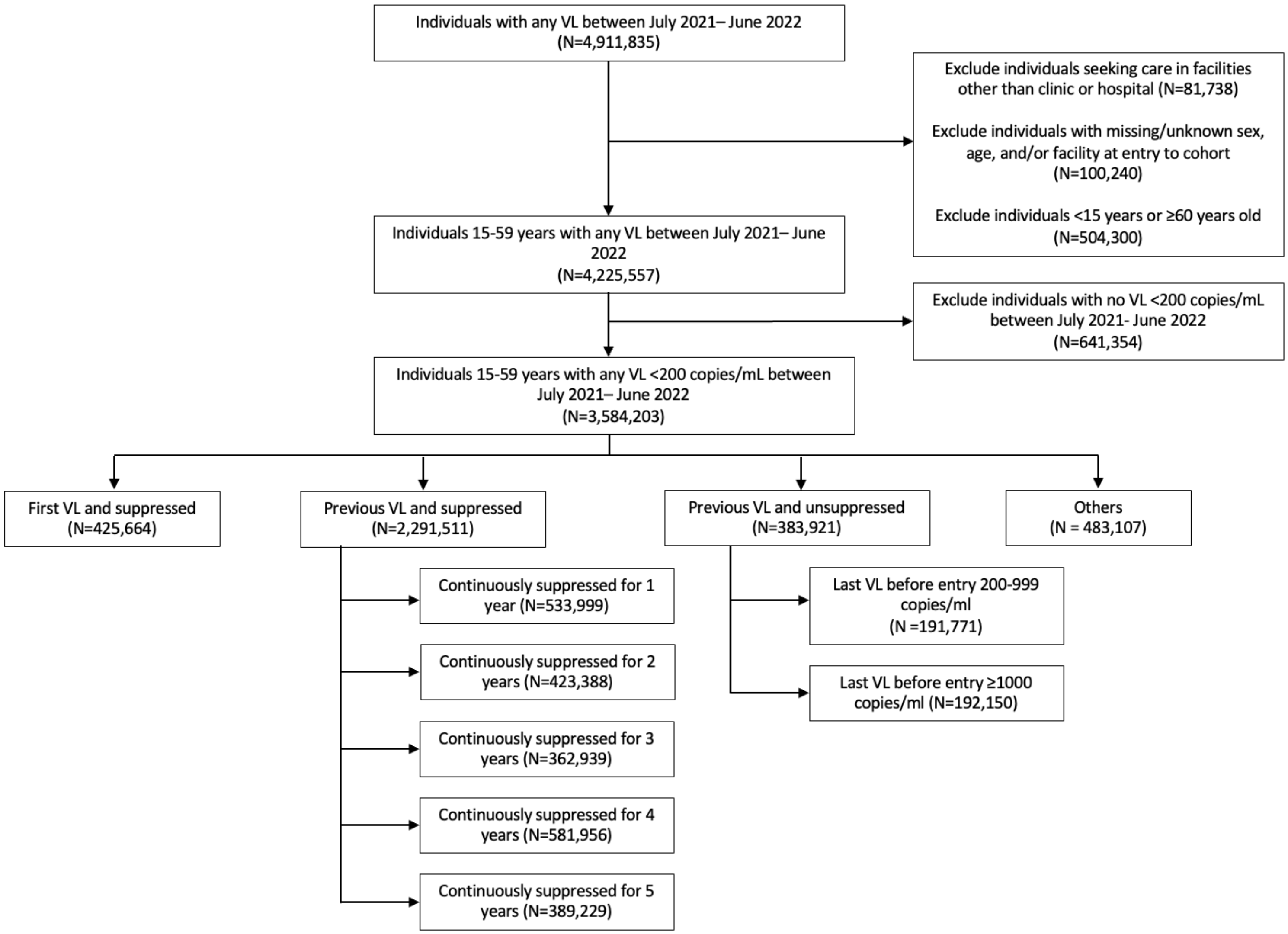

Note: see Table 1 note for viral load history definitions.

eFigure 2. Relative risk of (A) viral monitoring and (B) viral rebound at 12 months

Viral monitoring at 12-months

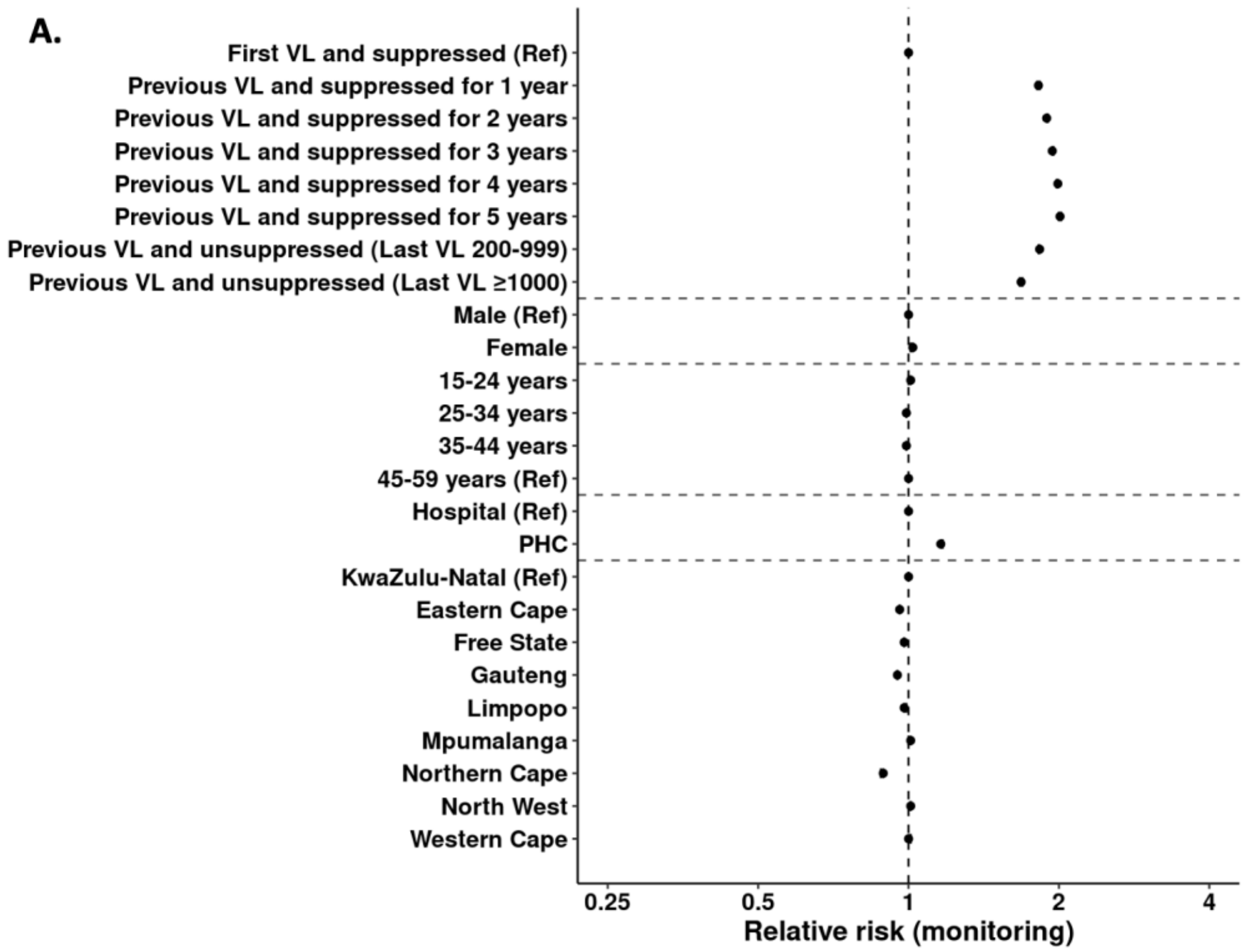

Viral rebound (>200 copies/mL) among those monitored at 12-months

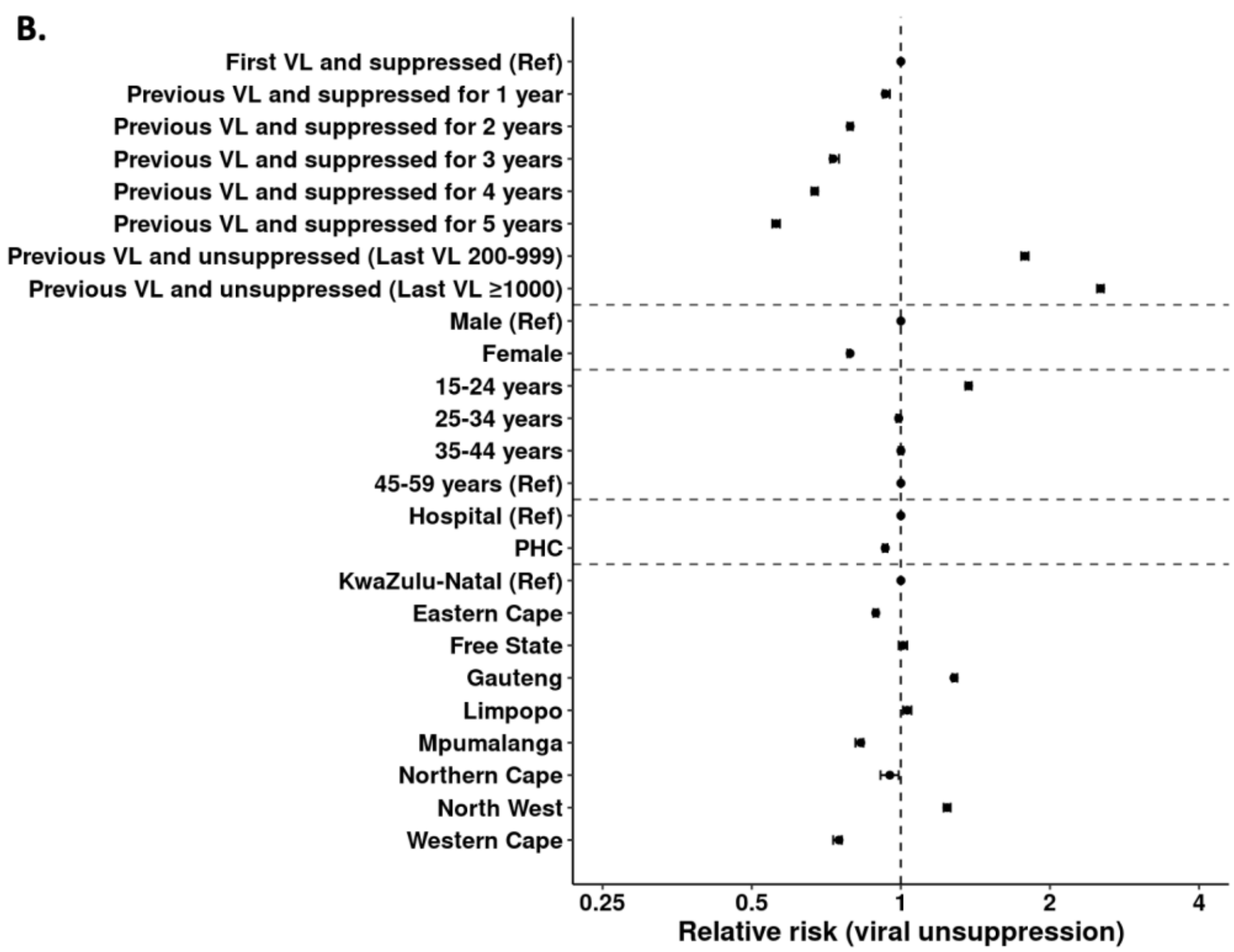

Note: see Table 1 note for viral load history definitions.
